# Natural Language Processing for Imaging Protocol Assignment: Machine Learning for Multiclass Classification of Abdominal CT Protocols Using Indication Text Data

**DOI:** 10.1007/s10278-022-00633-8

**Published:** 2022-06-02

**Authors:** Brian Arun Xavier, Po-Hao Chen

**Affiliations:** Imaging Institute, Cleveland Clinic Foundation, 9500 Euclid Ave., P34, Cleveland, OH 44195 USA

**Keywords:** Natural language processing, Machine learning, Deep learning, Protocol, Abdominal imaging

## Abstract

A correct protocol assignment is critical to high-quality imaging examinations, and its automation can be amenable to natural language processing (NLP). Assigning protocols for abdominal imaging CT scans is particularly challenging given the multiple organ specific indications and parameters. We compared conventional machine learning, deep learning, and automated machine learning builder workflows for this multiclass text classification task. A total of 94,501 CT studies performed over 4 years and their assigned protocols were obtained. Text data associated with each study including the ordering provider generated free text study indication and ICD codes were used for NLP analysis and protocol class prediction. The data was classified into one of 11 abdominal CT protocol classes before and after augmentations used to account for imbalances in the class sample sizes. Four machine learning (ML) algorithms, one deep learning algorithm, and an automated machine learning (AutoML) builder were used for the multilabel classification task: Random Forest (RF), Tree Ensemble (TE), Gradient Boosted Tree (GBT), multi-layer perceptron (MLP), Universal Language Model Fine-tuning (ULMFiT), and Google’s AutoML builder (Alphabet, Inc., Mountain View, CA), respectively. On the unbalanced dataset, the manually coded algorithms all performed similarly with F1 scores of 0.811 for RF, 0.813 for TE, 0.813 for GBT, 0.828 for MLP, and 0.847 for ULMFiT. The AutoML builder performed better with a F1 score of 0.854. On the balanced dataset, the tree ensemble machine learning algorithm performed the best with an F1 score of 0.803 and a Cohen’s kappa of 0.612. AutoML methods took a longer time for completion of NLP model training and evaluation, 4 h and 45 min compared to an average of 51 min for manual methods. Machine learning and natural language processing can be used for the complex multiclass classification task of abdominal imaging CT scan protocol assignment.

**Supplementary Information:**

The online version contains supplementary material available at 10.1007/s10278-022-00633-8.

## Introduction

Radiology utilization trends in the inpatient and outpatient settings are proliferating with the most substantial increase seen in CT scan volumes [1, 2]. Radiologists spend time reviewing study indications and the patient’s clinical history to assign appropriate protocols for each scan. An accurate protocol is critical to ensure imaging is performed on the correct body part, contrast is appropriately administered, and scanner parameters are tailored to the patient’s specific need [3]. Although some imaging indications are complex and benefit from active radiologist involvement, automating the protocol assignment of routine examinations may allow a radiologist to spend more time on other interpretive and non-interpretive tasks [4, 5].

Applying machine learning (ML) techniques in natural language processing (NLP) may alleviate the radiologist’s workload by automating the protocol assignment of routine imaging examinations [6]. Prior research has begun to investigate natural language processing for imaging protocol assignments. For instance, Lee et al. established that machine learning can be used to differentiate musculoskeletal MRI orders as “routine” or “tumor” examination using the patient’s age, gender, referring department, and indication [7]. Similarly, another study applied NLP towards musculoskeletal protocol classification to predict whether intravenous contrast was necessary for an MRI scan based on the clinical indication [8, 9]. However, compared with these binary classification tasks, protocolling often requires choosing between more than two possible options. In abdominal imaging, the complexity of indications and parameters for the multiple possible organ-specific pathologies requires selecting from many CT protocol choices.

Rapid developments in machine learning over the last decade prompted by hardware and software advancements have led to many algorithms and models to choose from. As the field has developed from experimental to producing top results in the real world, there has been a push to simplify and aid in implementing machine learning for the industry. Conventionally, ML tools range in complexity. Researchers can choose to build models entirely in code using programming languages such as Python with open-source libraries such as TensorFlow or PyTorch [10]. Alternatively, tools such as KNIME (Knime AG, Zurich, Switzerland) offer a drag-and-drop graphical interface [11]. These tools typically run on user-owned computer hardware.

The advent of cloud-based, automatic ML tools has decreased the barrier to entry for ML by automating the model selection and parameter-tuning as well as obviating the need for powerful on-premise hardware. Although these features can make ML more accessible to the radiologist, a paucity of literature directly compares automated ML approaches against conventional methods within medical imaging.

Therefore, we applied natural language processing to analyze radiology study indication text data for the multiclass classification task of abdominal CT protocol assignment. We also assessed the accuracy, ease-of-use, and time needed to build and run manually designed machine learning models compared to automated machine learning builders.

## Materials and Methods

### Gathering and Preprocessing the Protocol Data

The study was approved by the authors’ Institutional Review Board. From the radiology information system (RIS) (Syngo Workflow, Siemens Healthineers, Germany), abdominal imaging CT studies performed between 2016 and 2019 at our institution were identified. All study and final assigned protocol data were exported in a tabular format and initially cleaned and normalized using Excel (Microsoft, Redmond, Washington, USA). Pertinent text data such as the referring provider’s free text indication data-why the study was requested to be performed-and the associated ICD diagnosis code were concatenated into a single string column. All personal identifiers were stripped. As the automatic builder tool does not support the use of both structured and unstructured data elements for NLP, the date of the study, patient age, gender, and others were also removed to maintain comparability of the ML models’ performance. Duplicate studies with identical text columns were removed Table 1.

**Table 1:** Protocol dataset metrics

	**Unbalanced dataset**		**Balanced dataset**	
**Samples** (***n***)	94,501		229,450	
**Median words per sample**	12.0		12.0	
**Protocol classes**	11		11	
**Samples per Class**	(***n***)	(**%**)	(***n***)	(**%**)
Renal Stone	4145	4.39	20,000	9.1
Abdomen and Pelvis	66,448	70.3	20,000	9.1
Abdomen	4471	4.73	20,000	9.1
Pelvis	971	1.03	20,000	9.1
Kidney	2417	2.56	20,000	9.1
Urogram	5352	5.66	20,000	9.1
Cystogram	139	0.15	20,000	9.1
Pancreas	2086	2.21	20,000	9.1
Enterography	3733	3.95	20,000	9.1
Liver	3746	3.96	20,000	9.1
Adrenal	993	1.05	20,000	9.1
**Training set samples**	(*n*)	(%)	(*n*)	(%)
Machine learning and deep learning models	85,051	90.0	220,000	96.0
Automated deep learning builder	72,094	80.0	–––-	–––
**Validation Set Samples**	(*n*)	(%)	(*n*)	(%)
Machine learning and deep learning models	–––-	–––	–––-	–––
Automated deep learning builder	9012	10.0	–––-	–––
**Test set samples**	(*n*)	(%)	(*n*)	(%)
Machine learning and deep learning models	9450	10.0	9450	4.0
Automated deep learning builder	9012	10.0	–––-	–––

Thirty-three abdominal imaging CT protocols were grouped into 11 organ-specific protocol classes that matched the grouping presented on RIS. For example, all “CT Abdomen and Pelvis” protocols, including those with or without iv and oral contrast, were included under the “Abdomen and Pelvis” protocol class. Similarly, all “CT Liver” protocols including three and four-phase studies were included under the “Liver” protocol class. A complete list of protocol classes and the protocols they include are listed in Table 2. The final protocol performed for the study and assigned by the radiologist was used as the ground truth. The protocol class names were organized and substituted for an integer value based on a standardized key. Using this two-column format of the integer value of the assigned protocol class followed by the associated text information, the data was processed by each of the workflows separately.

**Table 2:** Abdominal CT protocol classes

**#**	**Protocol name**	**Protocol description(s)**
1	Renal stone	Acute flank pain/Renal stone protocol
2	Abdomen and pelvis	With IV and oral contrast; with IV, oral and rectal contrast; with IV and without oral contrast; without IV and with oral contrast; without iv and without oral contrast
3	Abdomen	With IV and oral contrast, with IV and without oral contrast, without IV and with oral contrast, without iv and without oral contrast
4	Pelvis	With IV and oral contrast; with IV, oral, and rectal contrast; without IV, with oral and rectal contrast
5	Kidney	Triple phase kidneys with pelvis, Triple phase kidneys without pelvis
6	Urogram	Triple phase urogram, Urogram with split bolus
7	Cystogram	Cystogram with IV contrast, Cystogram without IV contrast
8	Pancreas	Pancreas protocol with pelvis, Pancreas protocol without pelvis
9	Enterography	1-Phase Enterography, 2-Phase Enterography
10	Liver	2-Phase Liver protocol, 2-Phase Liver protocol with pelvis, 3-Phase Liver protocol, 3-Phase Liver protocol with pelvis, 4-Phase Liver protocol, 4-Phase Liver protocol with pelvis
11	Adrenal	Adrenal protocol with contrast, Adrenal protocol without contrast, Adrenalectomy protocol

To address data imbalance, a combination of undersampling, augmentation, and oversampling was used depending on the protocol class [12]. Random undersampling was applied to the largest class (Abdomen and Pelvis) to 20,000 samples. Text-based data augmentation and random oversampling was used in all other classes within the training data set to achieve data balance with 20,000 samples in all classes. All augmentations and sampling were performed only on the training set with the validation set remaining unmodified. Augmentation techniques used included back translation by translating the protocol indication text data to French and translating the output back to English to create minor modifications without loss of meaning [13]. Additional augmentations included replacing words with synonyms, randomly swapping words within a sample, and randomly deleting a word from a sample. The data was then processed by the manual workflows including through the four machine learning algorithms and the universal language model based deep learning algorithm. The data augmentation step is considered part of the data pre-processing steps for manual machine learning workflows. Therefore, only the original training dataset was submitted to the commercial automated machine learning builder.

### Manual Machine Learning Model Workflow

The free, open-source data analytics platform, KNIME, was used to preprocess further and evaluate the data using common NLP operations such as erasing punctuation, filtering stop words, and Porter stemming. The data was then converted to a two NGram bag-of-words model before vectorized using the inverse document frequency. Then, this preprocessed data was randomized and divided into training and testing sets before being input into four machine learning algorithms separately: random forest (RF), tree ensemble (TE), gradient boosted trees (GBT), and multi-layer perceptron (MLP) Table 3 [14, 15]. Random forest and tree ensemble algorithms were selected as examples of machine learning algorithms commonly used for classification tasks. Gradient boosted trees and mult-layer perceptrons were selected as algorithms sometimes shown to outperform random forests in ML tasks. The outputs of each algorithm were visualized as a confusion matrix and compared using precision, recall, and F1 scores. Class specific F1 scores and Cohen’s kappa were also calculated. The execution time of the text processing and each model’s training and inference time was recorded.

**Table 3:** Key model parameters

**Name**	**Value**
**Random forest**	
Number of models	64
Split criterion	Gini Index
**Tree ensemble**	
Split crterion	Gini Index
**Gradient boosted trees**	
Tree depth limit	4
Number of models	100
Learning rate	0.1
**Multi-layer perceptron**	
Algorithm name	RProp
Activation function	Logistic
Number of hidden layers	1
Number of hidden neurons per layer	10
Maximum number of iterations	100
**Universal language model** **fine-tuning**	
Learning parameters	
Loss function	Categorical Cross Entropy
Optimizer	Adam
Training parameters	
Architecture	AWD-LSTM
Learning rate	2E-2
Epochs	10
Batch size	128
Dropout rate	0.1
**Automated deep learning builder**	
Architecture	*N/A

^*^Parameters for automated deep learning builders are not accessible

Additionally, a deep-learning language model workflow was also deployed. The deidentified two-column data was processed using Python within a Jupyter Notebook instance. The primary libraries used included SpaCY for text preprocessing and the Fast. AI deep learning library for training and fine-tuning of a language model for the classification task. Similar to the KNIME workflow, the data processing included shuffling the data for randomness, dividing it into training, testing, and validation datasets, and removing any data with empty columns. Using a previously described ULMFiT technique and AWD-LSTM model, a pre-trained general language model was loaded containing approximately 103 million words obtained from the 28,595 Wikipedia articles in the Wikitext-103 corpus [16–18]. Further fine-tuning of the language model was performed on the entirety of the CT protocol dataset, which trained the model to predict the next word of the dataset. After fine-tuning, the pre-trained weights and biases of the model were used to aide in the performance of the original task of classifying the given text field into one of the 11 abdominal CT protocol classes. An AWD-LSTM was again used for the classification task, and training was performed until the validation loss was minimized. FastAI automatically incorporated multiple state-of-the-art paradigms for efficient and effective training, including an optimal learning rate finder, variable learning rates throughout training, and dropout. The results, including loss values, F1 scores, and Cohen’s kappa, were obtained, and a confusion matrix was created for visual analysis. Execution time, including processing, training, and validation, was evaluated using Python’s standard library.

### Automated Machine Learning Builder Workflow

We imported the same two-column text data to Google’s AutoML automated commercial builder platform (Alphabet, Inc., Mountain View, CA) to create an NLP classification model. The 11 classes and individual samples were presented for a brief, final edit, including moving a sample to a different class or deleting a sample. After reviewing, the training and inference of the dataset were initiated through a single button click. Several hours later, a second notification was received by e-mail that the process was complete. Results, including precision, accuracy, and F1 score, were available to view as well as a confusion matrix and a live interface for testing the model on new text data if desired. Finally, data processing time and model training time was calculated for comparison.

## Results

After processing the entries, including removing new and duplicate orders, 94,501 abdominal CT studies performed between 12/30/2015 and 09/15/2019 were included for evaluation in the unbalanced dataset (Table 1). The automated ML builder required more stringent removal of duplicates, including those across various labels resulting in 90,118 entries for evaluation. The balanced dataset created using modifications such as oversampling and data augmentation had 229,450 total samples.

**Fig. 1:**
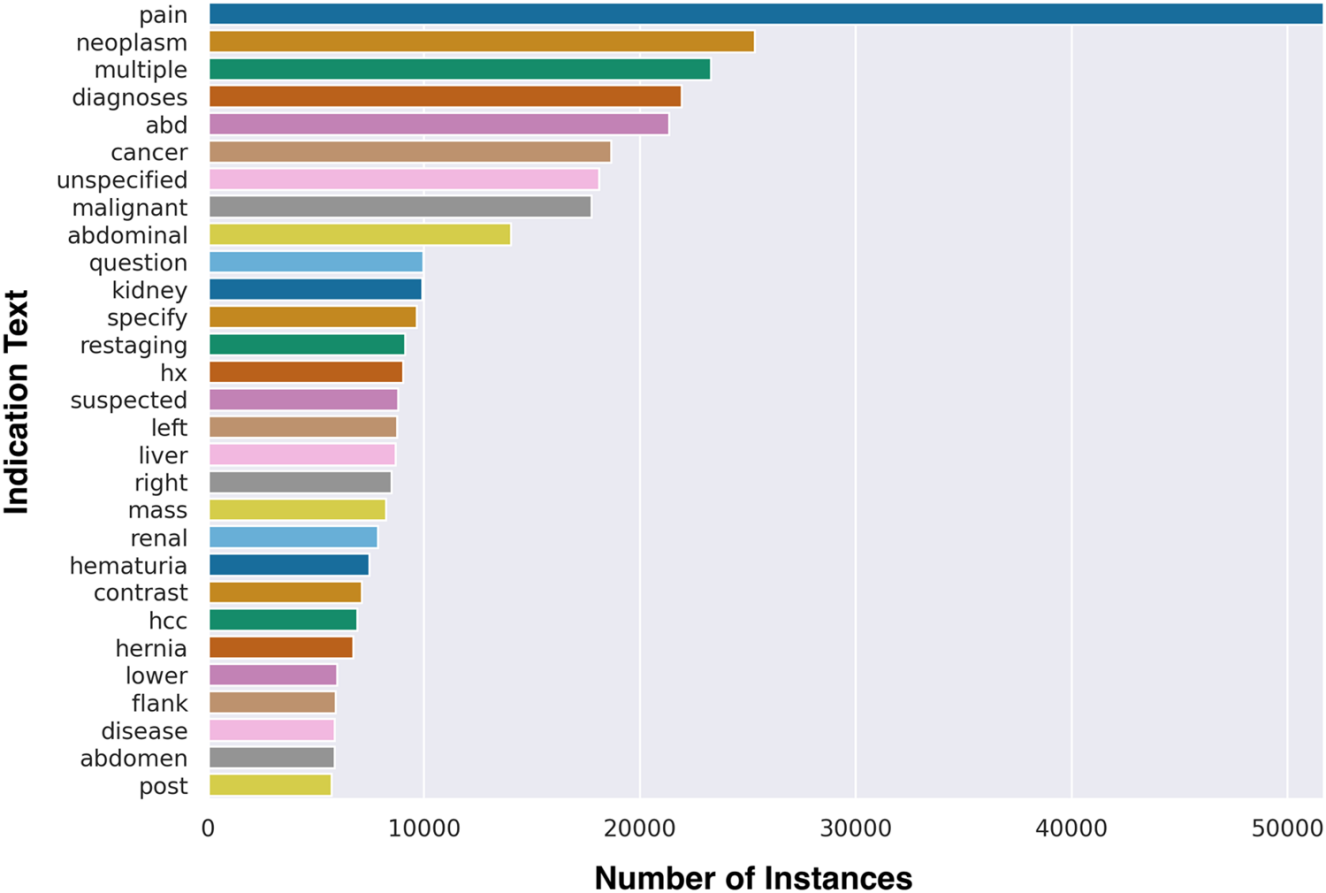
Frequency distribution of words in indication text data

**Fig. 2:**
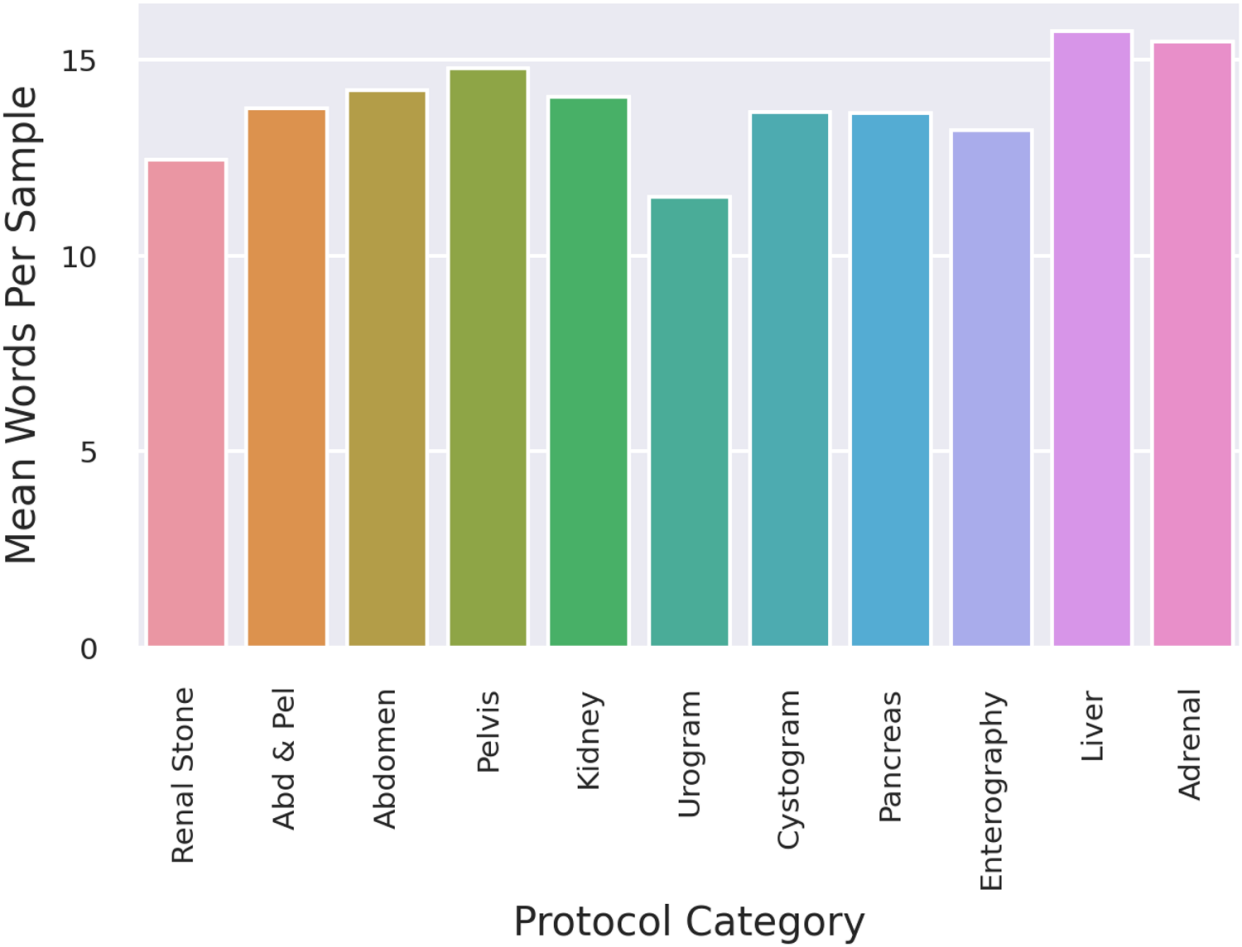
Mean words per sample by protocol category

Total analysis of all studies performed demonstrated that the most common words included in the text data included “multiple diagnoses,” “pain,” and “neoplasm” with greater than 15,000 instances each (Fig. 1). The mean words per sample were 14, which remained similar across all protocol classes (Fig. 2).

On the unbalanced dataset, the highest F1 score was achieved by the automated ML builder at 0.854, with a recall of 83.9% and 87.0% at a confidence threshold of 0.5 Table 4. The manually coded language model-based deep learning algorithm, ULMFiT, achieved an F1 score of 0.847 and a Cohen’s kappa score of 0.679. The four machine learning algorithms performed similarly with an F1 score of 0.811 for the random forest, 0.813 for the tree ensemble, 0.813 for the gradient boosted trees, and 0.828 for the multi-layer perceptron.

**Table 4:** Model results comparison

	**Unbalanced dataset**		**Balanced dataset**	
**Model name**	**F1 score**	**Cohen’s Kappa**	F1 score**F1 score**	**Cohen’s Kappa**
Random forest, Knime	0.811	0.537	0.799	0.604
Tree ensemble, Knime	0.813	0.542	0.803	0.612
Gradient boosted trees, Knime	0.813	0.556	0.746	0.551
Multi-layer perceptron, Knime	0.828	0.601	0.678	0.482
Universal language model, Python	0.847	0.679	0.765	0.584
Automated machine learning builder	0.854	0.678	-	-

On the balanced dataset with 20,000 samples for all classes, the tree ensemble machine learning algorithm performed the best with an F1 score of 0.803 and a Cohen’s kappa of 0.612. The random forest performed second best with a F1 score of 0.799 and Cohen’s kappa of 0.604. The ULMFiT deep learning algorithm achieved an F1 score of 0.765 and a Cohen’s kappa score of 0.584. Class specific F1 scores for both unbalanced and balanced datasets are included in the supplemental data.

**Fig. 3:**
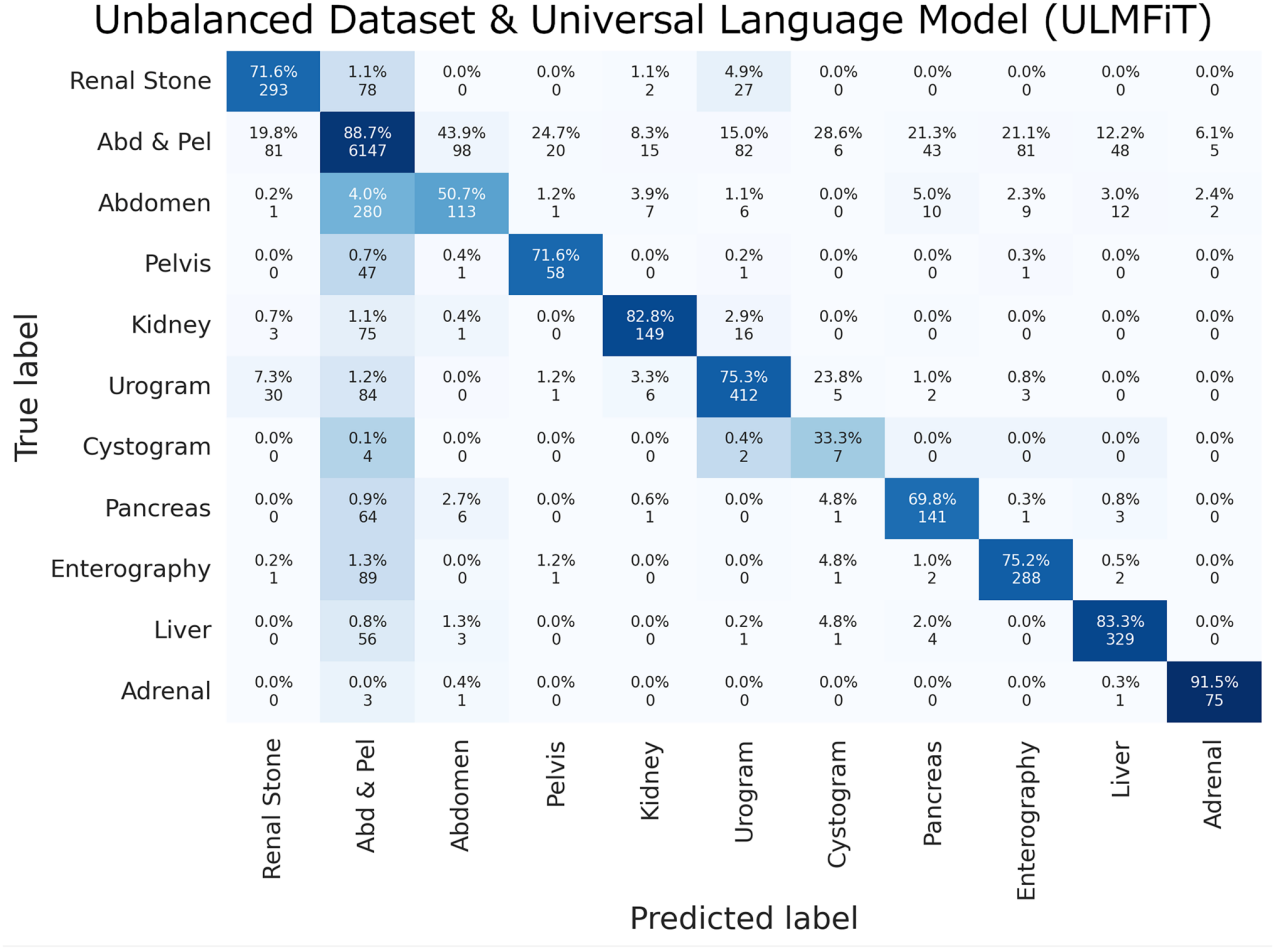
Unbalanced dataset and universal language model (ULMFiT) confusion matrix

**Fig. 4:**
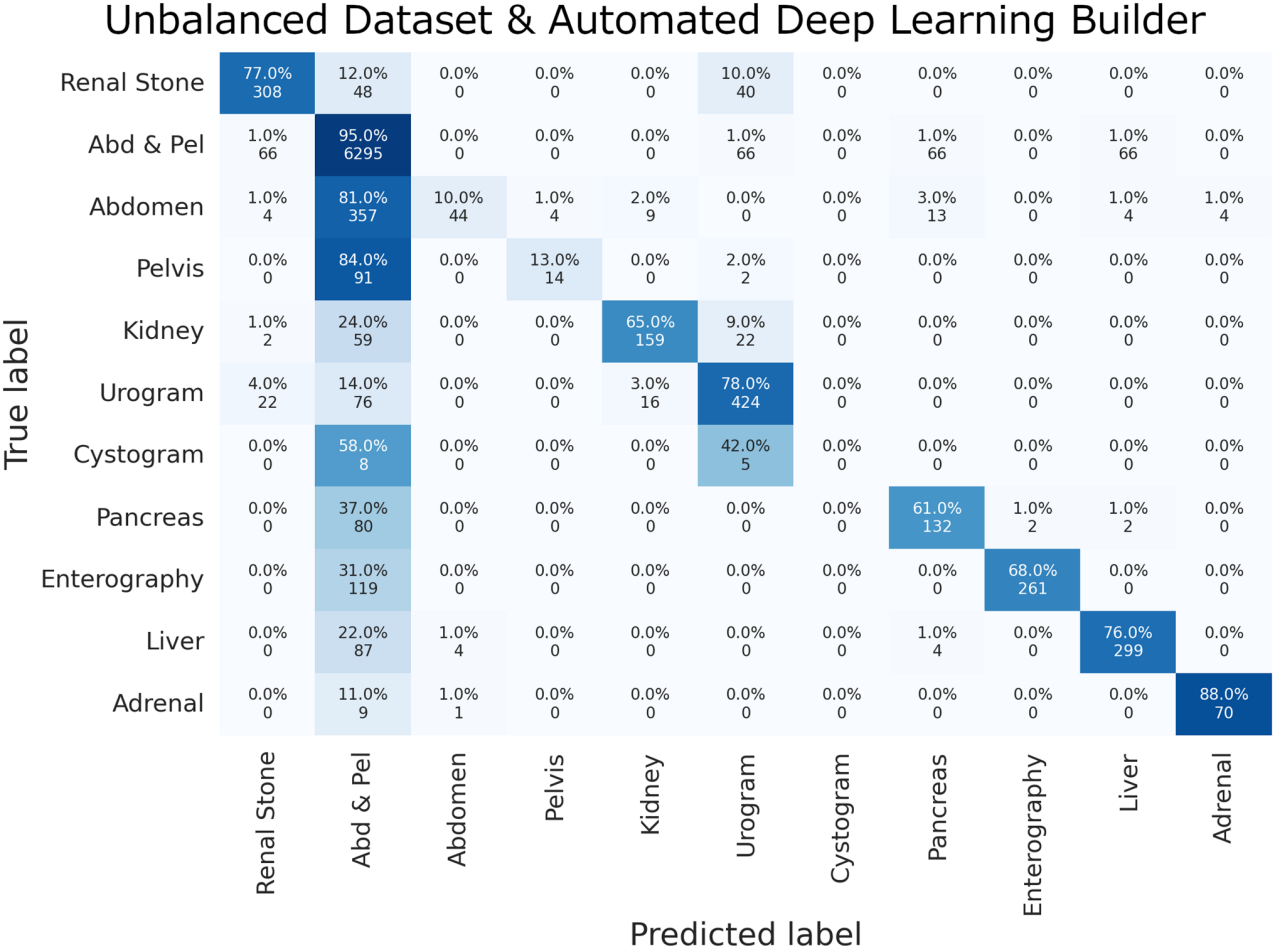
Unbalanced dataset and automated deep learning builder confusion matrix

**Fig. 5:**
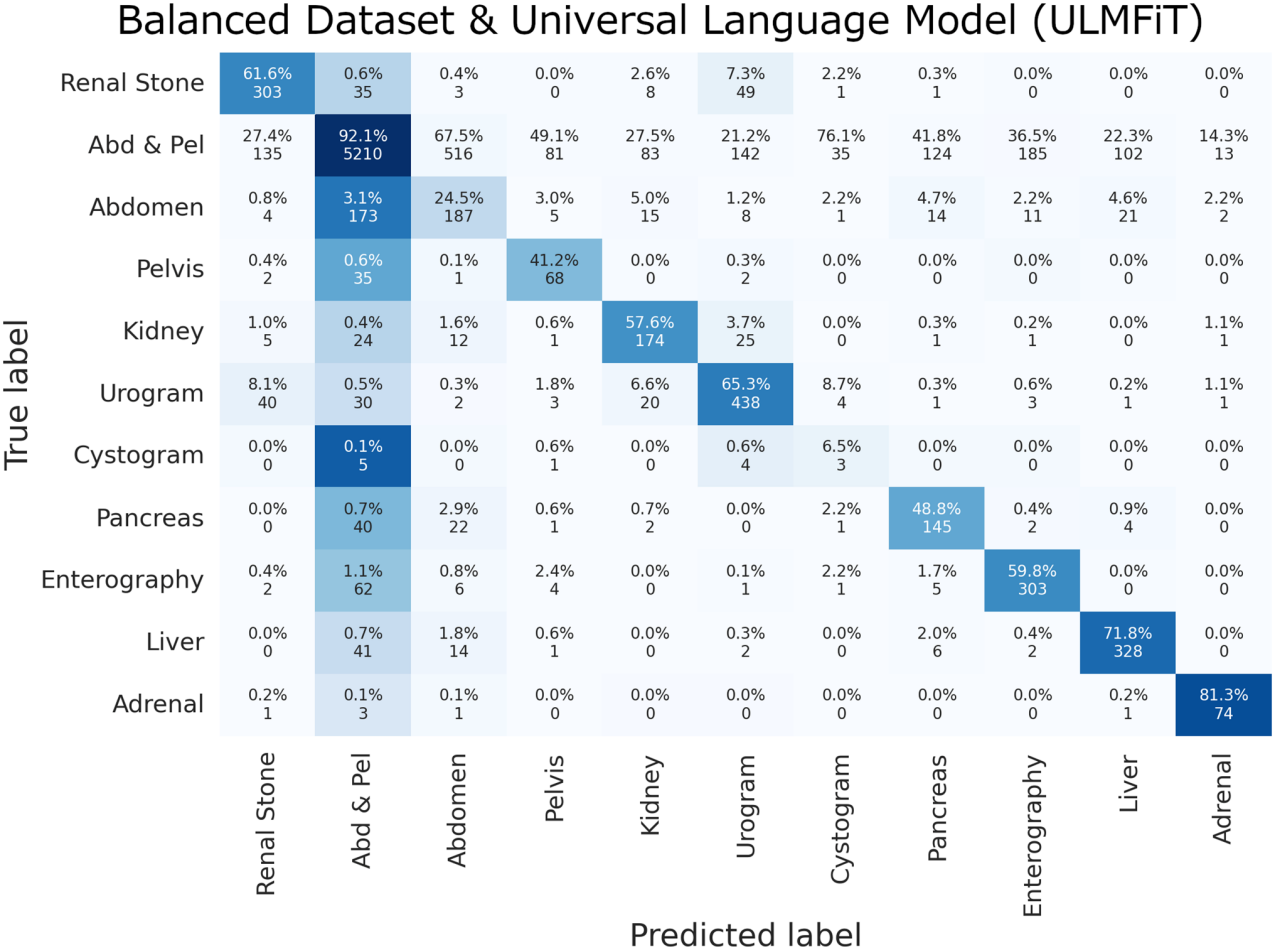
Balanced dataset and universal language model (ULMFiT) confusion matrix

The top protocol classes that were most often classified correctly included the “Abdomen and Pelvis” class and the “Adrenal” protocol class. The top misclassified protocol classes were “Abdomen” only, “Cystogram”, and “Pelvis” only classes, most often labeled as the “Abdomen and Pelvis” protocol class. Examples of the universal language model’s most incorrect predictions in the validation set are listed in Table 6 by identifying the largest loss values and probability estimates - a surrogate for the algorithm’s confidence in protocol classification. Complete confusion matrices for the manually coded deep learning model and the automated ML builder are displayed in Figs. 3, 4, and 5.

Objective measurements of each workflow’s text processing and model training and evaluation time are listed in Table 5, along with the authors’ subjective assessment of the difficulty of producing the complete workflow end-to-end and hardware cost for the project. Although the automated ML builder achieved a slightly higher F1 score, the text processing time and model training time were 77 and 29 times longer than for the ULMFiT model, respectively.

**Table 5:** Model production time and difficulty

**Model Name**	**Text preprocessing time**	**Model training and evaluation time**	**Inference time**	**User expertise**	**Hardware**
Random forest, Knime	39 min	16 min	Negligible	+ +	Owned/local
Tree ensemble, Knime	39 min	30 min	Negligible	+ +	Owned/local
Gradient boosted trees, Knime	39 min	2 h 57 min	Negligible	+ +	Owned/local
Multi-layer perceptron, Knime	39 min	22 min	Negligible	+ +	Owned/local
Universal language model, Python	21 s	10 min	Negligible	+ + + +	Owned/local
Automated machine learning builder	27 min	4 h 45 min	Negligible	+	Leased/cloud

## Discussion

### Approach to Protocol Assignment

Assigning an imaging protocol (i.e., “protocoling”) is a key part of the radiology workflow but a difficult problem to optimize for the informatics specialist [19]. At many institutions and practices, protocoling imaging examinations is a human-driven process. Our work suggests that NLP can be valuable in assisting the human expert in this task. However, existing work in protocol assignment using NLP have been limited in the number of protocol classes addressed. For instance, Trivedi et al. used 1520 studies to train and test a binary classification algorithm separating MRI examinations requiring versus those not requiring gadolinium-based intravenous contrast [8]. Lee et al. performed an analysis using 6276 studies to train and test a binary classification algorithm assigning “routine” versus “tumor” protocol classes [7].

Limiting the number of protocol classes and including only commonly used imaging protocols avoids some of the challenges seen in real life. Many protocols in radiology exist to address uncommon indications, leading to data imbalance problems. When multiple protocols are considered simultaneously, a model’s performance must also be considered at a class-specific level. Our work adds to the existing literature in three ways. First, we leveraged a larger dataset of 94,510 abdominal imaging studies and increased the number of simultaneously evaluated protocol classes addressing the problem as a multiclass classification task instead of a binary one. Additionally, our work also highlights the challenges - and possible solutions - to data imbalance and class-specific performance that must be addressed in text classification. Finally, the present work compares different machine learning approaches to show their respective trade-offs.

Data imbalance is a critical problem for multiclass classification problems in NLP and ML. In the present study, some of the classes such as CT cystogram are underutilized relative to routine CT of the abdomen and pelvis by several orders of magnitude as the dataset included all exams performed between two timepoints. Anecdotally, the present dataset is representative of a typical radiology practice in that a small number of protocols represent the vast majority of examinations performed. The largest class is more than one-100- fold more abundant than the smallest class and also substantially larger than other minority classes by approximately one order of magnitude.

### Managing Class-Imbalanced Datasets

Data imbalance machine learning problems in radiology are primarily studied using pixel-based data [20]. In radiology text processing and NLP, data imbalance has been highlighted as a source of problem [12]. However, to the authors’ knowledge, the present study is one of the first that discusses the effectiveness of mitigation strategies in radiology including model performance with and without data rebalancing efforts. In the present study, several strategies were employed, including random undersampling of the majority class, random oversampling of the minority classes, and using data augmentation techniques such as random word modifications and back translation to generate similar text indication data [12, 13]. Aggressive oversampling can produce an overfitted model that generalizes poorly to unseen data, whereas overly aggressive undersampling reduces the complexity that could be captured in a larger dataset. Our results agree with the published literature that data augmentation techniques such as back translation can be effective for high-dimensional imbalanced dataset [13]. Our experience using cross-class performance measures including Cohen’s kappa and label-specific performance measures suggest that a combined augmentation/oversampling and undersampling approach yielded a slightly worse performance by overall F1 metrics but, in return, achieved substantially improved performance for minority classes. Nevertheless, our data shows that augmentation aids but does not eliminate problems for protocols with very few native samples; for instance, accurately identifying the “Cystogram” class remained a challenge for all models.

### Comparing Machine Learning Approaches

Overall performances by the manually coded machine learning and deep learning techniques were comparable to the automated machine learning builder with the best performance by the ULMFiT and automated ML models on the unbalanced dataset and the tree ensemble and random forest models on the balanced dataset. All models and methods demonstrated similar, predictable classification errors with the most difficulty classifying the protocols with the least samples, as detailed in the previous section.

The decision of which technique to use depends on multiple factors, including the informatics specialist’s NLP knowledge, desire for customization, and resources such as hardware and time. Manually coded methods allow for flexibility in model development, improvement, and scaling both pre- and post-production. With the support of graphical drag-and-drop software or common NLP coding libraries, these methods are becoming quicker and more intuitive for all users. In contrast, automated ML builders provide an accessible interface and perform much of the data engineering such as the NLP preprocessing, feature selection, dimensionality reduction, training, and cross-validation of the model. However, these simplifications come at the cost of customizability and transparency with limited control of all aspects of the NLP pipeline. The cost profile of these approaches also differ. Whereas manually coded methods require informaticists to deploy the software suites on self-sourced server hardware, the automatic builder couples the cost of the hardware and software and operates entirely on the cloud.

It also should be noted that augmentation strategies such as back translation and oversampling are only possible with either the fully manual or the drag-and-drop methods. The automatic ML builder provided no meaningful tool to address class-imbalanced datasets, with the documentation recommending that including more training data for the minority class is the best solution. While acquiring more training data is theoretically the best solution, it is not always possible or practical. Therefore, despite achieving an overall high performance, the automatic ML model demonstrates a bias against minority class labels; for instance, the automatic ML builder model appears to ignore the cystogram protocol. While the manually coded ULMFiT and traditional ML models may demonstrate comparable or even lower F1-scores, the effort towards rebalancing the dataset are reflected in the stronger label-specific performance measures. While ultimate choice depends on the clinical use case, the present study demonstrates the need to compare across multiple ML approaches to understand the downstream effects of data imbalance.

### Limitations and Future Directions

Although additional factors exist that limit the applicability of our work, it is one step towards tackling the real-world complexity of protocol assignment. Future studies would attempt to classify more specific protocols within each organ system across a broader patient population. Similarly, the models should be tested on broader text data and use cases in radiology to ensure that their general understanding of medical language persists. As demonstrated in Table 6, in some cases, the actual exam performed was perhaps less appropriate than the predicted protocol when using only the indication text. Alternatively, multiple protocols could have been considered appropriate, whereas the model is graded on the assignment of a single “correct” protocol. Determining a protocol class from the indication text data alone can be challenging, and the actual exam protocol may have been chosen only after additional chart review by the radiologist. Future studies could address this by creating a dedicated ground truth for ML model training, combining multiple radiologist’s class assignment using only the indication text compared with the algorithm’s. Additionally, we hope to include the clinical context such as the ordering provider’s name, the ordering department, and the clinical notes leading to the imaging order into future NLP models, expecting them to improve performance similar to how they often aid the human expert in the protocoling process.

**Table 6:** Top 10 incorrect predictions by universal language model (ULMFiT)

**Protocol indication text**	**Target**	**Predicted**
“Placenta accreta, third trimester Other (specify in question below) Intentional surgical cystotomy for placenta accreta Placenta accreta, third trimester”	7, Cystogram	2, Abdomen & Pelvis
“Abdominal mass, unspecified abdominal location Mass or lump, abdomen pelvis pelvic mass”	4, Pelvis	2, Abdomen & Pelvis
“multiple diagnoses previous differentiate left UPJ obstruction versus left parapelvic renal cyst hematuria”	7, Cystogram	6, Urogram
“Other (specify in question below) r/o bleed post IR procedure”	8, Pancreas	2, Abdomen & Pelvis
“Constipation, unspecified Other (specify in question below) abdominal distention and constipation”	4, Pelvis	2, Abdomen & Pelvis
“Secondary malignant neoplasm of bone Pt with unknown primary (poorly undifferentiated carcinoma) with metastasis to bone. Please evaluate for staging and possible identification of primary. Pelvic Pain CT A/P 8/17/16”	4, Pelvis	2, Abdomen & Pelvis
“Other (specify in question below) Abdominal pain. History of small bowel obstruction”	4, Pelvis	2, Abdomen & Pelvis
“Pt s/p ERCP, narrowed CBD visualized, wonder if any intra-pancreatic mass compression on BD Obstruction of bile duct, Pt s/p ERCP, narrowed CBD visualized, wonder if any intra-pancreatic mass comp”	8, Pancreas	2, Abdomen & Pelvis
“H/O PROSTATECTOMY. NOW WITH SUSPECTED FLUID-FILLED LYMPH NODES”	4, Pelvis	2, Abdomen & Pelvis
“Benign carcinoid tumor, unspecified site Elevated Chromogranin!, evaluate for Possible Pancreatic NET Benign neuroendocrine tumors Benign carcinoid tumor, unspecified site”	10, Liver	8, Pancreas

## Supplementary Information

Below is the link to the electronic supplementary material.

Supplementary file1 (DOCX 44 KB)

## Data Availability

N/A.

## Abbreviations

AI: Artificial intelligence
ML: Machine learning
NLP: Natural language processing
RF: Random forest
TE: Tree ensemble
GBT: Gradient boosted tree
MLP: Multi-layer perceptron
ULMFiT: Universal language model fine-tuning
AutoML: Automated machine learning

## Code Availability

N/A.

## References

[1] Selvarajan SK, Levin DC, Parker L: The Increasing Use of Emergency Department Imaging in the United States: Is It Appropriate? 10.2214/AJR.19.21386. American Roentgen Ray Society. 213(4):W180–W184, 2019. 10.2214/AJR.19.21386. 10.2214/AJR.19.21386 31237433

[2] McDonald RJ, Schwartz KM, Eckel LJ, et al: The Effects of Changes in Utilization and Technological Advancements of Cross-Sectional Imaging on Radiologist Workload. Academic Radiology. Elsevier 22(9):1191–1198,2015. 10.1016/J.ACRA.2015.05.007. 10.1016/j.acra.2015.05.007 26210525

[3] Bell LTO, James R, Rosa JA, Pollentine A, Pettet G, McCoubrie P: Reducing interruptions during duty radiology shifts, assessment of its benefits and review of factors affecting the radiology working environment. Clinical Radiology. W.B. Saunders 73(8):759.e19–759.e25,2018. 10.1016/J.CRAD.2018.04.007. 10.1016/j.crad.2018.04.007 29853302

[4] Kansagra AP, Liu K, Yu JPJ: Disruption of radiologist workflow. Current problems in diagnostic radiology. Mosby 45(2):101–106,2016. 10.1067/J.CPRADIOL.2015.05.006. 10.1067/j.cpradiol.2015.05.006 26122926

[5] Lee MH, Schemmel AJ, Pooler BD, et al: Workflow dynamics and the imaging value chain: Quantifying the effect of designating a nonimage-interpretive task workflow. current problems in diagnostic radiology. Mosby 46(4):275–281,2017. 10.1067/J.CPRADIOL.2016.11.010. 10.1067/j.cpradiol.2016.11.010 28049559

[6] Chen PH: Essential elements of natural language processing: What the radiologist should know. Academic Radiology. Elsevier 27(1):6–12,2020. 10.1016/J.ACRA.2019.08.010. 10.1016/j.acra.2019.08.010 31537505

[7] Lee YH: Efficiency improvement in a busy radiology practice: Determination of musculoskeletal magnetic resonance imaging protocol using deep-learning convolutional neural networks. J Digit Imaging. Springer New York LLC. 31(5):604–610,2018. 10.1007/S10278-018-0066-Y/TABLES/5. 10.1007/s10278-018-0066-y PMC6148815 29619578

[8] Trivedi H, Mesterhazy J, Laguna B, Vu T, Sohn JH: Automatic Determination of the Need for Intravenous Contrast in Musculoskeletal MRI Examinations Using IBM Watson’s Natural Language Processing Algorithm. J Digit Imaging. Springer New York LLC 31(2):245–251,2018. 10.1007/S10278-017-0021-3/TABLES/4. 10.1007/s10278-017-0021-3 PMC5873465 28924815

[9] Ferrucci D, Levas A, Bagchi S, Gondek D, Mueller ET (2013). Watson: Beyond Jeopardy! Artificial Intelligence. Elsevier.

[10] Chatley R, Donaldson A, Mycroft A: The Next 7000 Programming Languages. Lecture Notes in Computer Science (including subseries Lecture Notes in Artificial Intelligence and Lecture Notes in Bioinformatics). Springer, Cham 10000:250–282,2019. 10.1007/978-3-319-91908-9_15.

[11] Berthold MR, Cebron N, Dill F, et al: KNIME - the Konstanz information miner. ACM SIGKDD Explorations Newsletter. ACM PUB27 New York, NY, USA; 58–61, 2009. 10.1145/1656274.1656280.

[12] Olthof AW, van Ooijen PMA, Cornelissen LJ: Deep learning-based natural language processing in radiology: The impact of report complexity, disease prevalence, dataset size, and algorithm type on model performance. J Med Syst. Springer; 45(10):1–16,2021. 10.1007/S10916-021-01761-4/TABLES/6. 10.1007/s10916-021-01761-4 PMC8416876 34480231

[13] Ayoub J, Yang XJ, Zhou F: Combat COVID-19 infodemic using explainable natural language processing models. Information Processing & Management. Elsevier 58(4):102569,2021. 10.1016/J.IPM.2021.102569. 10.1016/j.ipm.2021.102569 PMC7980090 33776192

[14] Friedman JH: Greedy function approximation: A gradient boosting machine. The Annals of Statistics. Institute of Mathematical Statistics 29(5):1189–1232,2001. http://www.jstor.org/stable/2699986.

[15] Wang Y, Sohn S, Liu S, et al: A clinical text classification paradigm using weak supervision and deep representation. BMC Med Inform Decis Mak. BioMed Central Ltd 19(1):1–13, 2019. 10.1186/S12911-018-0723-6/FIGURES/4. 10.1186/s12911-018-0723-6 PMC6322223 30616584

[16] Merity S, Xiong C, Bradbury J, Socher R: Pointer Sentinel Mixture Models. 5th International Conference on Learning Representations, ICLR 2017 - Conference Track Proceedings. International Conference on Learning Representations, ICLR, 2016. https://arxiv.org/abs/1609.07843v1. Accessed February 10, 2022.

[17] Merity S, Keskar NS, Socher R: Regularizing and Optimizing LSTM Language Models. 6th International Conference on Learning Representations, ICLR 2018 - Conference Track Proceedings. International Conference on Learning Representations, ICLR, 2017. https://arxiv.org/abs/1708.02182v1. Accessed February 10, 2022.

[18] Howard J, Ruder S: Universal Language Model Fine-tuning for Text Classification. ACL 2018 - 56th Annual Meeting of the Association for Computational Linguistics, Proceedings of the Conference (Long Papers). Association for Computational Linguistics (ACL) 1:328–339,2018. 10.18653/v1/p18-1031.

[19] Protocol Design and Optimization | American College of Radiology. . https://www.acr.org/Practice-Management-Quality-Informatics/Imaging-3/Imaging-Value-Chain/Protocol-Optimization. Accessed February 10, 2022.

[20] Dong Q, Gong S, Zhu X: Imbalanced deep learning by minority class incremental rectification. IEEE Transactions on Pattern Analysis and Machine Intelligence. IEEE Computer Society 41(6):1367–1381,2019. 10.1109/TPAMI.2018.2832629. 10.1109/TPAMI.2018.2832629 29993438

