# Supplementary material for: Natural Language Processing for Imaging Protocol Assignment: Machine Learning for Multiclass Classification of Abdominal CT Protocols Using Indication Text Data
Source: J Digit Imaging. 2022 Jun 2;35(5):1120–30. doi: 10.1007/s10278-022-00633-8 (PMC9582109; doi:10.1007/s10278-022-00633-8)
Supplement: Supplementary file 1 — Supplementary file1 (DOCX 44 KB) [file 10278_2022_633_MOESM1_ESM.docx]

**Supplemental Data**

**Table A1: Unbalanced Dataset Category Specific Results – Random Forest**

|  | **True Positive** | **False Positive** | **True Negative** | **False Negative** | **Precision** | **Recall** | **F1-score** |
| --- | --- | --- | --- | --- | --- | --- | --- |
| **Renal Stone** | 242 | 107 | 8,943 | 158 | 60.5% | 69.3% | 0.646 |
| **Abdomen and Pelvis** | 6,427 | 1,454 | 1,370 | 199 | 97.0% | 81.6% | 0.886 |
| **Abdomen** | 21 | 14 | 8,995 | 420 | 4.8% | 60.0% | 0.088 |
| **Pelvis** | 9 | 0 | 9,342 | 99 | 8.3% | 100.0% | 0.154 |
| **Kidney** | 124 | 31 | 9,175 | 120 | 50.8% | 80.0% | 0.622 |
| **Urogram** | 292 | 95 | 8,812 | 251 | 53.8% | 75.5% | 0.628 |
| **Cystogram** | 0 | 0 | 9,437 | 13 | 0.0% |  |  |
| **Pancreas** | 72 | 26 | 9,207 | 145 | 33.2% | 73.5% | 0.457 |
| **Enterography** | 163 | 16 | 9,050 | 221 | 42.4% | 91.1% | 0.579 |
| **Liver** | 252 | 35 | 9,021 | 142 | 64.0% | 87.8% | 0.740 |
| **Adrenal** | 63 | 7 | 9,363 | 17 | 78.8% | 90.0% | 0.840 |

**Table A2: Unbalanced Dataset Category Specific Results – Tree Ensemble**

|  | **True Positive** | **False Positive** | **True Negative** | **False Negative** | **Precision** | **Recall** | **F1-score** |
| --- | --- | --- | --- | --- | --- | --- | --- |
| **Renal Stone** | 249 | 99 | 8,951 | 151 | 62.3% | 71.6% | 0.666 |
| **Abdomen and Pelvis** | 6,428 | 1,446 | 1,378 | 198 | 97.0% | 81.6% | 0.887 |
| **Abdomen** | 25 | 8 | 9,001 | 416 | 5.7% | 75.8% | 0.105 |
| **Pelvis** | 7 | 3 | 9,339 | 101 | 6.5% | 70.0% | 0.119 |
| **Kidney** | 123 | 30 | 9,176 | 121 | 50.4% | 80.4% | 0.620 |
| **Urogram** | 291 | 101 | 8,806 | 252 | 53.6% | 74.2% | 0.622 |
| **Cystogram** | 0 | 0 | 9,437 | 13 | 0.0% |  |  |
| **Pancreas** | 73 | 23 | 9,210 | 144 | 33.6% | 76.0% | 0.466 |
| **Enterography** | 163 | 17 | 9,049 | 221 | 42.4% | 90.6% | 0.578 |
| **Liver** | 259 | 38 | 9,018 | 135 | 65.7% | 87.2% | 0.750 |
| **Adrenal** | 62 | 5 | 9,365 | 18 | 77.5% | 92.5% | 0.844 |

**Table A3: Unbalanced Dataset Category Specific Results – Gradient Boosted Trees**

|  | **True Positive** | **False Positive** | **True Negative** | **False Negative** | **Precision** | **Recall** | **F1-score** |
| --- | --- | --- | --- | --- | --- | --- | --- |
| **Renal Stone** | 255 | 105 | 8,945 | 145 | 63.8% | 70.8% | 0.671 |
| **Abdomen and Pelvis** | 6,343 | 1322 | 1,502 | 283 | 95.7% | 82.8% | 0.888 |
| **Abdomen** | 33 | 27 | 8,982 | 408 | 7.5% | 55.0% | 0.132 |
| **Pelvis** | 16 | 13 | 9,329 | 92 | 14.8% | 55.2% | 0.234 |
| **Kidney** | 126 | 40 | 9,166 | 118 | 51.6% | 75.9% | 0.615 |
| **Urogram** | 303 | 105 | 8,802 | 240 | 55.8% | 74.3% | 0.637 |
| **Cystogram** | 1 | 2 | 9,435 | 12 | 7.7% | 33.3% | 0.125 |
| **Pancreas** | 84 | 33 | 9,200 | 133 | 38.7% | 71.8% | 0.503 |
| **Enterography** | 202 | 50 | 9,016 | 182 | 52.6% | 80.2% | 0.635 |
| **Liver** | 261 | 51 | 9,005 | 133 | 66.2% | 83.7% | 0.739 |
| **Adrenal** | 63 | 15 | 9,355 | 17 | 78.8% | 80.8% | 0.797 |

**Table A4: Unbalanced Dataset Category Specific Results – Multi-layer Perceptron**

|  | **True Positive** | **False Positive** | **True Negative** | **False Negative** | **Precision** | **Recall** | **F1-score** |
| --- | --- | --- | --- | --- | --- | --- | --- |
| **Renal Stone** | 272 | 130 | 8,920 | 128 | 68.0% | 67.7% | 0.678 |
| **Abdomen and Pelvis** | 6,353 | 1187 | 1,637 | 273 | 95.9% | 84.3% | 0.897 |
| **Abdomen** | 2 | 2 | 9,007 | 439 | 0.5% | 50.0% | 0.009 |
| **Pelvis** | 14 | 5 | 9,337 | 94 | 13.0% | 73.7% | 0.220 |
| **Kidney** | 130 | 50 | 9,156 | 114 | 53.3% | 72.2% | 0.613 |
| **Urogram** | 366 | 116 | 8,791 | 177 | 67.4% | 75.9% | 0.714 |
| **Cystogram** | 0 | 0 | 9,437 | 13 | 0.0% |  |  |
| **Pancreas** | 108 | 44 | 9,189 | 109 | 49.8% | 71.1% | 0.585 |
| **Enterography** | 218 | 28 | 9,038 | 166 | 56.8% | 88.6% | 0.692 |
| **Liver** | 293 | 53 | 9,003 | 101 | 74.4% | 84.7% | 0.792 |
| **Adrenal** | 66 | 13 | 9,357 | 14 | 82.5% | 83.5% | 0.830 |

**Table A5: Unbalanced Dataset Category Specific Results – Universal Language Model**

|  | **True Positive** | **False Positive** | **True Negative** | **False Negative** | **Precision** | **Recall** | **F1-score** |
| --- | --- | --- | --- | --- | --- | --- | --- |
| **Renal Stone** | 301 | 99 | 8,919 | 131 | 75.3% | 69.7% | 0.724 |
| **Abdomen and Pelvis** | 6,094 | 532 | 2,081 | 743 | 92.0% | 89.1% | 0.905 |
| **Abdomen** | 105 | 336 | 8,907 | 102 | 23.8% | 50.7% | 0.324 |
| **Pelvis** | 67 | 41 | 9,302 | 40 | 62.0% | 62.6% | 0.623 |
| **Kidney** | 152 | 92 | 9,150 | 56 | 62.3% | 73.1% | 0.673 |
| **Urogram** | 434 | 109 | 8,744 | 163 | 79.9% | 72.7% | 0.761 |
| **Cystogram** | 2 | 11 | 9,430 | 7 | 15.4% | 22.2% | 0.182 |
| **Pancreas** | 144 | 73 | 9,170 | 63 | 66.4% | 69.6% | 0.679 |
| **Enterography** | 285 | 99 | 8,984 | 82 | 74.2% | 77.7% | 0.759 |
| **Liver** | 318 | 76 | 8,983 | 73 | 80.7% | 81.3% | 0.810 |
| **Adrenal** | 74 | 6 | 9,356 | 14 | 92.5% | 84.1% | 0.881 |

**Table A6: Balanced Dataset Category Specific Results – Random Forest**

|  | **True Positive** | **True Negative** | **False Positive** | **False Negative** | **Precision** | **Recall** | **F1-score** |
| --- | --- | --- | --- | --- | --- | --- | --- |
| **Renal Stone** | 305 | 183 | 8,867 | 95 | 62.5% | 76.3% | 0.687 |
| **Abdomen and Pelvis** | 5,727 | 716 | 2,108 | 899 | 88.9% | 86.4% | 0.876 |
| **Abdomen** | 116 | 225 | 8,784 | 325 | 34.0% | 26.3% | 0.297 |
| **Pelvis** | 39 | 43 | 9,299 | 69 | 47.6% | 36.1% | 0.411 |
| **Kidney** | 170 | 148 | 9,058 | 74 | 53.5% | 69.7% | 0.605 |
| **Urogram** | 395 | 206 | 8,701 | 148 | 65.7% | 72.7% | 0.691 |
| **Cystogram** | 7 | 13 | 9,424 | 6 | 35.0% | 53.8% | 0.424 |
| **Pancreas** | 141 | 108 | 9,125 | 76 | 56.6% | 65.0% | 0.605 |
| **Enterography** | 255 | 114 | 8,952 | 129 | 69.1% | 66.4% | 0.677 |
| **Liver** | 320 | 133 | 8,923 | 74 | 70.6% | 81.2% | 0.756 |
| **Adrenal** | 71 | 15 | 9,355 | 9 | 82.6% | 88.8% | 0.855 |

**Table A7: Balanced Dataset Category Specific Results – Tree Ensemble**

|  | **True Positive** | **True Negative** | **False Positive** | **False Negative** | **Precision** | **Recall** | **F1-score** |
| --- | --- | --- | --- | --- | --- | --- | --- |
| **Renal Stone** | 309 | 198 | 8,852 | 91 | 77.3% | 60.9% | 0.681 |
| **Abdomen and Pelvis** | 5,760 | 699 | 2,125 | 866 | 86.9% | 89.2% | 0.880 |
| **Abdomen** | 119 | 214 | 8,795 | 322 | 27.0% | 35.7% | 0.307 |
| **Pelvis** | 37 | 40 | 9,302 | 71 | 34.3% | 48.1% | 0.400 |
| **Kidney** | 184 | 138 | 9,068 | 60 | 75.4% | 57.1% | 0.650 |
| **Urogram** | 391 | 199 | 8,708 | 152 | 72.0% | 66.3% | 0.690 |
| **Cystogram** | 6 | 12 | 9,425 | 7 | 46.2% | 33.3% | 0.387 |
| **Pancreas** | 143 | 107 | 9,126 | 74 | 65.9% | 57.2% | 0.612 |
| **Enterography** | 249 | 108 | 8,958 | 135 | 64.8% | 69.7% | 0.672 |
| **Liver** | 319 | 132 | 8,924 | 75 | 81.0% | 70.7% | 0.755 |
| **Adrenal** | 71 | 15 | 9,355 | 9 | 88.8% | 82.6% | 0.855 |

**Table A8: Balanced Dataset Category Specific Results – Gradient Boosted Trees**

|  | **True Positive** | **True Negative** | **False Positive** | **False Negative** | **Precision** | **Recall** | **F1-score** |
| --- | --- | --- | --- | --- | --- | --- | --- |
| **Renal Stone** | 305 | 183 | 8,867 | 95 | 62.5% | 76.3% | 68.7% |
| **Abdomen and Pelvis** | 5,727 | 716 | 2,108 | 899 | 88.9% | 86.4% | 87.6% |
| **Abdomen** | 116 | 225 | 8,784 | 325 | 34.0% | 26.3% | 29.7% |
| **Pelvis** | 39 | 43 | 9,299 | 69 | 47.6% | 36.1% | 41.1% |
| **Kidney** | 170 | 148 | 9,058 | 74 | 53.5% | 69.7% | 60.5% |
| **Urogram** | 395 | 206 | 8,701 | 148 | 65.7% | 72.7% | 69.1% |
| **Cystogram** | 7 | 13 | 9,424 | 6 | 35.0% | 53.8% | 42.4% |
| **Pancreas** | 141 | 108 | 9,125 | 76 | 56.6% | 65.0% | 60.5% |
| **Enterography** | 255 | 114 | 8,952 | 129 | 69.1% | 66.4% | 67.7% |
| **Liver** | 320 | 133 | 8,923 | 74 | 70.6% | 81.2% | 75.6% |
| **Adrenal** | 71 | 15 | 9,355 | 9 | 82.6% | 88.8% | 85.5% |

**Table A9: Balanced Dataset Category Specific Results – Multi-layer Perceptron**

|  | **True Positive** | **True Negative** | **False Positive** | **False Negative** | **Precision** | **Recall** | **F1-score** |
| --- | --- | --- | --- | --- | --- | --- | --- |
| **Renal Stone** | 335 | 323 | 8,727 | 65 | 83.8% | 50.9% | 0.633 |
| **Abdomen and Pelvis** | 4,437 | 402 | 2,422 | 2,189 | 67.0% | 91.7% | 0.774 |
| **Abdomen** | 180 | 904 | 8,105 | 261 | 40.8% | 16.6% | 0.236 |
| **Pelvis** | 76 | 303 | 9,039 | 32 | 70.4% | 20.1% | 0.312 |
| **Kidney** | 184 | 195 | 9,011 | 60 | 75.4% | 48.5% | 0.591 |
| **Urogram** | 343 | 175 | 8,732 | 200 | 63.2% | 66.2% | 0.647 |
| **Cystogram** | 8 | 45 | 9,392 | 5 | 61.5% | 15.1% | 0.242 |
| **Pancreas** | 166 | 218 | 9,015 | 51 | 76.5% | 43.2% | 0.552 |
| **Enterography** | 269 | 245 | 8,821 | 115 | 70.1% | 52.3% | 0.599 |
| **Liver** | 330 | 195 | 8,861 | 64 | 83.8% | 62.9% | 0.718 |
| **Adrenal** | 75 | 42 | 9,328 | 5 | 93.8% | 64.1% | 0.761 |

**Table A10: Balanced Dataset Category Specific Results – Universal Language Model**

|  | **True Positive** | **False Positive** | **True Negative** | **False Negative** | **Precision** | **Recall** | **F1-score** |
| --- | --- | --- | --- | --- | --- | --- | --- |
| **Renal Stone** | 303 | 97 | 8,861 | 189 | 75.8% | 61.6% | 0.679 |
| **Abdomen and Pelvis** | 5,210 | 1,416 | 2,376 | 448 | 78.6% | 92.1% | 0.848 |
| **Abdomen** | 187 | 254 | 8,432 | 577 | 42.4% | 24.5% | 0.310 |
| **Pelvis** | 68 | 40 | 9,245 | 97 | 63.0% | 41.2% | 0.498 |
| **Kidney** | 174 | 70 | 9,078 | 128 | 71.3% | 57.6% | 0.637 |
| **Urogram** | 438 | 105 | 8,674 | 233 | 80.7% | 65.3% | 0.722 |
| **Cystogram** | 3 | 10 | 9,394 | 43 | 23.1% | 6.5% | 0.102 |
| **Pancreas** | 145 | 72 | 9,081 | 152 | 66.8% | 48.8% | 0.564 |
| **Enterography** | 303 | 81 | 8,862 | 204 | 78.9% | 59.8% | 0.680 |
| **Liver** | 328 | 66 | 8,927 | 129 | 83.2% | 71.8% | 0.771 |
| **Adrenal** | 74 | 6 | 9,353 | 17 | 92.5% | 81.3% | 0.865 |
